# Analysis of mathematical model involving nonlinear systems of Caputo–Fabrizio fractional differential equation

**DOI:** 10.1186/s13661-023-01730-5

**Published:** 2023-04-19

**Authors:** Shiferaw Geremew Kebede, Assia Guezane Lakoud

**Affiliations:** 1 Mathematics Department, College of Natural Science, Arba Minch University, Arba Minch, Ethiopia; 2 Mathematics Department, Faculty of Sciences, Badji Mokhtar Annaba University, Annaba, Algeria

**Keywords:** 34A08, Caputo–Fabrizio fractional derivative, Fixed point theorems, Existence, Uniqueness, Stability of solution

## Abstract

In this paper, we consider a mathematical model of a coronavirus disease involving the Caputo–Fabrizio fractional derivative by dividing the total population into the susceptible population $\mathcal{S}(t)$, the vaccinated population $\mathcal{V}(t)$, the infected population $\mathcal{I}(t)$, the recovered population $\mathcal{R}(t)$, and the death class $\mathcal{D}(t)$. A core goal of this study is the analysis of the solution of a proposed mathematical model involving nonlinear systems of Caputo–Fabrizio fractional differential equations. With the help of Lipschitz hypotheses, we have built sufficient conditions and inequalities to analyze the solutions to the model. Eventually, we analyze the solution for the formed mathematical model by employing Krasnoselskii’s fixed point theorem, Schauder’s fixed point theorem, the Banach contraction principle, and Ulam–Hyers stability theorem.

## Introduction

Nowadays, various researchers have an intense interest in the mathematical construction of epidemic models and have suggested that the significance of a mathematical model for the purpose of examination and study is a modest method to deliberate the features of transferable diseases. However, as numerous researchers have demonstrated, a mathematical model emerges in many scientific and real-world problems, particularly nonlinear systems, and describing different natural phenomena using only differential equations of integer order is insufficient. Hence, in recent decades, different types of fractional derivatives, which are an extension of an integer order derivative to any random order derivative, have been employed for the designation and modification of various difficult solutions to several multifaceted real-life problems. Therefore, to overcome the difficulty that arises in the classic integer-order mathematical models, an extension of ordinary calculus was given by Leibniz and L’Hospital in 1695 [1], where integrals and derivatives are defined for arbitrary real order. Despite the fact that many research studies have shown that the application of the fractional derivative has gained significant acceptance and importance in a variety of widely used fields of science and epidemic problems, mathematical models are required to overcome the difficulties involved in differential equations of integer order. One can refer to [2] for the use of different types of fractional derivatives to model numerous epidemic problems mathematically.

Recently, a new fractional operator with a nonsingular kernel in its fractional derivatives without Gamma function was introduced by Caputo and Fabrizio (see [3]). The necessity of this type of fractional derivative is to express a class of nonlocal systems that cannot be defined by classical local theories or by fractional models with singular kernels. Moreover, this new operator is very useful for practical applications in the class of $C[a,b]$ (the space of continuous real-valued functions on the interval $[a,b]$) or $C^{1}[a,b]$ (the space of continuous differentiable functions on $[a,b]$), since the dynamic processes are smooth and have no discontinuities. Also, the furthermost sustaining character of this operator is that if we use the Laplace transformation, then any real power can be turned into an integer order. As a result, this property assists us in finding solutions to several associated problems. Thus, the Caputo–Fabrizio fractional derivative is employed in the investigation of various realistic mathematical models of epidemic problems [4].

In recent years, a lot of literature and research studies have suggested the importance of mathematical models to analyze and manage various types of infectious disease. To study and control the broad development of COVID-19, several applied mathematical analysts and researchers have mathematically modeled a number of infectious diseases, in particular COVID-19. One can refer to [5–7]. The authors in [8, 9] investigated a mathematical model of COVID-19 via the Atangana–Baleanu–Caputo ($\mathcal{ABC}$) fractional derivative with a nonsingular kernel. The susceptible–infected–recovered (SIR) model of the dynamical behavior of COVID-19 was studied in [10] by classifying the total population into the susceptible, exposed, infectious, recovered, the quarantine population, the recovered-exposed population, and the dead population. As a result, the authors investigated the stability of the equilibrium point, the theoretical effect of quarantine strategies, and numerical simulations of the model. The feast of the COVID-19 mathematical model was studied in the work [11] by portioning out the transmissibility of superspreader individuals and duplicate numbers, the stability of the disease-free equilibrium, and the sensitivity.

Many remarkable research studies point out that investigating the solutions of nonlinear differential equations that involve fractional derivatives by applying fixed point theorems is one of the most influential methods. In [12], the Krasnoselskii’s fixed point theorems and the Banach contraction principle were used to analyze the existence and uniqueness of solutions for a nonlinear system of fractional differential equations involving the Caputo–Hadamard fractional derivative. The fixed point theorems of Krasnoselskii and Banach were used to analyze solutions to differential equations involving nonsingular fractional derivatives in [13] and [14]. The Schauder’s and Mônch’s fixed point theorems and the technique of the measure of noncompactness were applied to investigate the solutions for a class of Caputo–Fabrizio fractional differential equations (see [15]). The analysis of solutions for nonlinear Caputo fractional differential equations using fixed point theorems based on Schauder’s fixed point theorem, the Banach contraction principle, and Krasnoselskii’s fixed point theorem was studied in [16]. The subject of the stability of functional equations was introduced in 1940 by Stanisaw Ulam, and the first substantial partial solution was provided in 1941 by D.H. Hyers. Following this, the Ulam–Hyers stability theorem was used by a number of authors to study the stability issues and can be applied to the solution analysis of a wide variety of fractional differential equations [17]. In [18], a family of generalized nonlinear fractional differential equations of order alpha ($1 < \alpha < 2$) were subjected to the Ulam–Hyers stability theorem. For solutions to fractional differential equations in the unit disk, [19] looked at the Hyers–Ulam stability for fractional differential equations in a complex Banach space. The existence, uniqueness, and Ulam–Hyers stability of solutions for nonlocal and multiple-point fractional boundary value problems in the framework of a generalized Hilfer derivative were studied in [20]. In the recent paper [21], the authors formulated a mathematical model of SIQR under the $\mathcal{ABC}$ fractional operator and analyzed its solution by using fixed point theorems. In [22], the qualitative analysis and stability in the occurrence of the basic reproduction number for the COVID-19 epidemic model with Atangana–Baleanu derivative have been studied. By employing Krasnoselskii’s and Banach fixed point theorems, the existence, uniqueness, and Ulam–Hyers stability result for BVPs of nonlinear fractional differential equations involving the generalized Caputo fractional derivative and Riemann–Liouville fractional integral boundary conditions were proven in [23]. In the research work [24], boundary value problems of nonlinear hybrid fractional differential equations through generalized Caputo operators have been studied by setting sufficient conditions for the existence of solutions by applying Dhage fixed point theorem for the sum of three operators. The existence and stability of fixed points, by introducing bifurcation theory and the corresponding numerical simulations for the complex dynamics of the Kopel model with nonsymmetric responses between oligopolists, Li, Liang, Shi, and He [25] investigated the existence of fold, transcritical, pitchfork, flip, and Neimark–Sacker bifurcations, from which various types of competition and cooperation between oligopolists emerge. By concentrating on the bifurcation analysis of a discrete-time Lotka–Volterra model utilizing a nonstandard finite difference discretization method, the paper [26] examined the demonstration of one interior fixed point in terms of its complicated dynamics. Li, Liang, and He [27] examined the existence of bifurcations, curve illustrations of fixed points, and one-parameter bifurcations with various periods for the multiperiodic dynamical behaviors of the planar Hindmarsh–Rose oscillator model.

Inspired by the above discussion, we consider, in this study, a system of nonlinear fractional derivative equations of the COVID-19 mathematical model involving the Caputo–Fabrizio fractional derivative by classifying epidemiological states of the total population based on individuals’ health status as follows: the susceptible population $\mathcal{S}(t)$, the vaccinated population $\mathcal{V}(t)$, the infected population $\mathcal{I}(t)$, the recovered population $\mathcal{R}(t)$, and the death class $\mathcal{D}(t)$ (death due to coronavirus or natural) at time *t*. Then the total population is

$$ \mathcal{N}(t)=\mathcal{S}(t)+\mathcal{V}(t)+\mathcal{I}(t)+ \mathcal{R}(t)+\mathcal{D}(t), $$

where $t\in I=[0,T]$, $\ T>0$. We investigate the solution of the modeled equation based on the arguments of fixed point theorems. To analyze the solution for the proposed model, we transform the equation into a fixed point problem, we build the required conditions to examine the existence, uniqueness and stability of solution by applying Banach contraction principle, Krasnoselskii’s fixed point theorem, Schauder’s fixed point theorem, and Ulam–Hyers stability theorem.

The primary goal of this study is to investigate the existence, uniqueness, and stability of the solution for the following systems of nonlinear fractional differential equations involving Caputo–Fabrizio fractional derivatives of order $\alpha \in (0,1)$, as shown in model (1) below:

1 $$ \textstyle\begin{cases} {}^{\mathcal{CF}}D_{0^{+}}^{\alpha }\mathcal{S}(t)=- ( \omega + \nu ) \mathcal{S}-\mu \mathcal{I}\mathcal{S,} \\ {}^{\mathcal{CF}}D_{0^{+}}^{\alpha }\mathcal{V}(t)=\nu \mathcal{S}- ( 1-\varepsilon ) \mu \mathcal{V,} \\ {}^{\mathcal{CF}}D_{0^{+}}^{\alpha }\mathcal{I}(t)=\mu \mathcal{S} \mathcal{I}- ( \eta +\omega ) \mathcal{I}, \\ {}^{\mathcal{CF}}D_{0^{+}}^{\alpha }\mathcal{R}(t)=\nu \mathcal{S}+ \eta \mathcal{I}, \\ {}^{\mathcal{CF}}D_{0^{+}}^{\alpha }\mathcal{D}(t)=\varpi \mathcal{I},\end{cases}$$

with the initial conditions

$$ \mathcal{S}(0)=\mathcal{S}_{0},\qquad \mathcal{V}(0)= \mathcal{V}_{0}, \qquad \mathcal{I}(0)=\mathcal{I}_{0},\mathcal{\qquad R}(0)=\mathcal{R}_{0}, \qquad \mathcal{D}(0)=\mathcal{D}_{0}, $$

where the parameters in the given model (1) are defined in Table 1.

**Table 1:** Description of the parameter described in the provided Model (1)

Notations	Description
*ν*	Vaccination rate
*ω*	Natural death rate
*ϖ*	Death rate in the infected class
*ε*	Infection reduction of vaccinated individuals
*μ*	Transmission rate of disease
*η*	Recovery rate

This research study is organized as follows: In Sect. 2, the required fixed point theorems are stated, and the necessary fractional operators are defined with their properties. Section 3 concerns the main results, which analyze the solution of a formulated mathematical model of COVID-19 involving the Caputo–Fabrizio fractional derivative. Furthermore, the existence, uniqueness, and stability of solutions are proved by applying fixed point theorems. This work ends with a conclusion and a list of some interesting articles.

## Preliminaries

The necessary fractional differential operators and their properties are given in this section. Additionally, we state some basic fixed point theorems and spaces that are needed to analyze the stability, existence and uniqueness of solution.

### Definition 2.1

([13])

If *X* is a Banach space, then $E\subset C(X)$ is equicontinuous if $\forall \epsilon >0$, $\exists \delta >0$, $\forall x,y\in X$:

$$ \Vert x-y \Vert < \delta \quad \Longrightarrow \quad \bigl\Vert T(x)-T(y) \bigr\Vert < \epsilon ,\quad \forall T\in E. $$

### Definition 2.2

([13])

A map $T:X\rightarrow X$ is completely continuous if the set $T(E) $ is relatively compact for any bounded subset *E* of *X*.

### Theorem 2.1

(Arzela–Ascoli theorem [13])

*Let* *X* *be a compact space*. *If* *E* *is an equicontinuous and bounded subset of* $C(X)$, *then the operator* *E* *is relatively compact*.

### Theorem 2.2

(Schauder’s fixed point theorem [13])

*Assume that* $(X,d)$ *is a complete metric space and* *D* *is a closed convex subset of* *X*. *If the map* $T:D\rightarrow D$ *is relatively compact*, *then the operator* *T* *has at least one fixed point* $x^{\ast }\in D$ *such that*

2 $$ Tx^{\ast }=x^{\ast }. $$

### Theorem 2.3

(Krasnoselskii’s fixed point theorem [28])

*Assume that* *D* *is a nonempty closed convex subset of a Banach space* *X*, $\mathcal{L}$ *and* $\mathcal{K}$ *are two operators that map* *D* *into* *X* *such that*:

1. $\mathcal{L}v+\mathcal{K}z\in D$, $\forall v,z\in D$,
2. $\mathcal{L}$ *is a contraction*, *and*
3. $\mathcal{K}$ *is compact and continuous*.

*Then*, $\mathcal{L}v+\mathcal{K}v=v$ *has at least one solution*.

### Definition 2.3

([29])

If $f\in H^{1}(a,b)=\{f:f\in L^{2}(a,b) \text{ and } f^{\prime }\in L^{2}(a,b)\}$ and $\beta \in {}[ 0,1]$, then the Caputo–Fabrizio fractional derivative is defined as

3 $$ {}^{\mathcal{CF}}D_{a}^{\beta } \bigl( f(t) \bigr) = \frac{\mathcal{M}(\beta )}{1-\beta } \int _{a}^{t}f^{\prime }(\tau )\exp \biggl[ -\beta \frac{t-\tau }{1-\beta } \biggr] \,d\tau , $$

where $\mathcal{M}(\alpha )$ is a normalization function with the property that $\mathcal{M}(0)=\mathcal{M}(1)=1$.

According to Definition 2.3, when $f(t) $ is constant, ${}^{\mathcal{CF}}D_{a}^{\beta }$ is zero, but the kernel does not have a singularity for $t=\tau $.

### Definition 2.4

([29])

The definition in equation (3) can be written for any $\beta \in {}[ 0,1] $ and any $f\in L^{1}(-\infty ,b)$ as

$$ {}^{\mathcal{CF}}D_{-\infty }^{\beta } \bigl( f(t) \bigr) = \frac{\beta \mathcal{M}(\beta )}{1-\beta } \int _{-\infty }^{t} \bigl( f(t)-f(\tau ) \bigr) \exp \biggl[ -\beta \frac{t-\tau }{1-\beta } \biggr] \,d\tau . $$

Losada and Nieto improved the Caputo–Fabrizio fractional derivative as follows:

4 $$ {}^{\mathcal{CF}}D_{a}^{\beta } \bigl( f(t) \bigr) = \frac{(2-\beta )\mathcal{M}(\beta )}{2(1-\beta )} \int _{a}^{t}f^{\prime }(\tau )\exp \biggl[ - \beta \frac{t-\tau }{1-\beta } \biggr] \,d\tau . $$

Also, Losada and Nieto defined the fractional integral corresponding to the derivative in equation (4) as follows.

### Definition 2.5

Let $0<\beta <1$. The fractional Caputo–Fabrizio integral of order *β* of a function *f* is defined by

5 $$ {}^{\mathcal{CF}}I_{0}^{\beta } \bigl( f(t) \bigr) = \frac{2(1-\beta )}{(2-\beta )\mathcal{M}(\beta )}f(t)+ \frac{2\beta }{(2-\beta )M(\beta )}\int _{0}^{t}f(\tau )\,d\tau ,\quad t\geq 0. $$

### Remark 2.1

The fractional integral of the Caputo–Fabrizio type of a function *f* of order $0<\beta <1$ is a mean between the function *f* and its integral of order one, according to Eq. (5),

6 $$ \frac{2(1-\beta )}{(2-\beta )\mathcal{M}(\alpha )}+ \frac{2\beta }{(2-\beta )\mathcal{M}(\beta )}=1, $$

and therefore $\mathcal{M}(\beta )=\frac{2}{2-\beta }$, $0<\beta <1$.

Losada and Nieto also proposed a new Caputo–Fabrizio derivative and its corresponding integral using $M(\beta )=\frac{2}{2-\beta }$.

### Definition 2.6

([29])

Let $0<\beta <1$. The fractional Caputo–Fabrizio derivative of order *β* of a function *f* is given by

7 $$ {}^{\mathcal{CF}}D_{0}^{\beta } \bigl( f(t) \bigr) = \frac{1}{1-\beta }\int _{0}^{t}f^{\prime }(\tau )\exp \biggl[ -\beta \frac{t-\tau }{1-\beta } \biggr] \,d\tau ,\quad t\geq 0, $$

and its corresponding fractional integral is defined as

8 $$ {}^{\mathcal{CF}}I_{0}^{\beta } \bigl( f(t) \bigr) =(1-\beta )f(t)+ \beta \int _{0}^{t}f(\tau )\,d\tau ,\quad t\geq 0, $$

such that

9 $$ \bigl( {}^{\mathcal{CF}}I_{0}^{\beta } \bigr) \bigl({}^{ \mathcal{CF}}D_{0}^{\alpha } \bigl( f(t) \bigr) \bigr) =f(t)-f(0). $$

The fundamental difference between the Caputo–Fabrizio operator and the old Caputo operator is that the new kernel has no singularity for $t= \tau $.

### Lemma 2.1

([30])

*The initial value problem*

10 $$ \textstyle\begin{cases} {}^{\mathcal{CF}}D_{0^{+}}^{\beta }u(t)=\phi (t),&t\geq 0, 0< \beta < 1, \\ u(0)=u_{0}, \end{cases}$$

*has a solution given by*

11 $$ u(t)=u_{0}+\frac{2(1-\beta )}{(2-\beta )\mathcal{M}(\beta )}\bigl(\phi (t)- \phi (0)\bigr)+\frac{2\beta }{(2-\beta )\mathcal{M}(\beta )} \int _{0}^{t} \phi (\tau )\,d\tau . $$

## Main results

In this section, we investigate the existence of at least one solution, the uniqueness, and the stability of the solution for the model (1). Let us begin by defining notations that are useful for our theorems. Denote

$$ X= \bigl\{ u\in C \bigl( [0,T],\mathbb{R}^{5} \bigr) : \Vert u \Vert < \infty \bigr\} $$

the Banach space with the norm

$$ \Vert u \Vert = \bigl\Vert (\mathcal{S},\mathcal{V},\mathcal{I}, \mathcal{R},\mathcal{D}) \bigr\Vert =\max_{t\in {}[ 0,T]} \bigl\{ \bigl\vert \mathcal{S}(t) \bigr\vert + \bigl\vert \mathcal{V}(t) \bigr\vert + \bigl\vert \mathcal{I}(t) \bigr\vert + \bigl\vert \mathcal{R}(t) \bigr\vert + \bigl\vert \mathcal{D}(t) \bigr\vert \bigr\} . $$

Rewrite the right-hand side of (1) as follows:

12 $$ \textstyle\begin{cases} \phi _{1}(t,u(t))=- ( \omega +\nu ) \mathcal{S}-\mu \mathcal{I}\mathcal{S,} \\ \phi _{2}(t,u(t))=\nu \mathcal{S}- ( 1-\varepsilon ) \mu \mathcal{V,} \\ \phi _{3}(t,u(t))=\mu \mathcal{S}\mathcal{I}- ( \eta +\omega ) \mathcal{I}, \\ \phi _{4}(t,u(t))=\nu \mathcal{S}+\eta \mathcal{I}, \\ \phi _{5}(t,u(t))=\varpi \mathcal{I}.\end{cases}$$

By applying Lemma 2.1 to equation (1) and using (12), we obtain the following integral equations:

$$\begin{aligned}& \mathcal{S}(t) =\mathcal{S}_{0}+ \frac{2(1-\alpha )}{(2-\alpha )M(\alpha )} \bigl[ \phi _{1}\bigl(t,u(t)\bigr)-\phi _{1}\bigl(0,u(0) \bigr) \bigr] + \frac{2\alpha }{(2-\alpha )M(\alpha )} \int _{0}^{t}\phi _{1}\bigl(\tau ,u( \tau )\bigr)\,d\tau , \\& \mathcal{V}(t) =\mathcal{V}_{0}+ \frac{2(1-\alpha )}{(2-\alpha )M(\alpha )} \bigl[ \phi _{2}\bigl(t,u(t)\bigr)-\phi _{2}\bigl(0,u(0) \bigr) \bigr] + \frac{2\alpha }{(2-\alpha )M(\alpha )} \int _{0}^{t}\phi _{2}\bigl(\tau ,u( \tau )\bigr)\,d\tau , \\& \mathcal{I}(t) =\mathcal{I}_{0}+ \frac{2(1-\alpha )}{(2-\alpha )M(\alpha )} \bigl[ \phi _{3}\bigl(t,u(t)\bigr)-\phi _{3}\bigl(0,u(0) \bigr) \bigr] + \frac{2\alpha }{(2-\alpha )M(\alpha )} \int _{0}^{t}\phi _{3}\bigl(\tau ,u( \tau )\bigr)\,d\tau , \\& \mathcal{R}(t) =\mathcal{R}_{0}+ \frac{2(1-\alpha )}{(2-\alpha )M(\alpha )} \bigl[ \phi _{4}\bigl(t,u(t)\bigr)-\phi _{4}\bigl(0,u(0) \bigr) \bigr] + \frac{2\alpha }{(2-\alpha )M(\alpha )} \int _{0}^{t}\phi _{4}\bigl(\tau ,u( \tau )\bigr)\,d\tau , \\& \mathcal{D}(t) =\mathcal{D}_{0}+ \frac{2(1-\alpha )}{(2-\alpha )M(\alpha )} \bigl[ \phi _{5}\bigl(t,u(t)\bigr)-\phi _{5}\bigl(0,u(0) \bigr) \bigr] + \frac{2\alpha }{(2-\alpha )M(\alpha )} \int _{0}^{t}\phi _{5}\bigl(\tau ,u( \tau )\bigr)\,d\tau . \end{aligned}$$

Denote

$$\begin{aligned}& w_{0} = ( \mathcal{S}_{0},\mathcal{V}_{0}, \mathcal{I}_{0}, \mathcal{R}_{0},\mathcal{D}_{0} ) , \\& w(t) = \bigl( \mathcal{S}(t),\mathcal{V}(t),\mathcal{I}(t), \mathcal{R}(t),\mathcal{D}(t) \bigr), \end{aligned}$$

and

$$ \Phi \bigl(t,w(t)\bigr)= \bigl( \phi _{1}\bigl(t,w(t)\bigr),\phi _{2}\bigl(t,w(t)\bigr),\phi _{3}\bigl(t,w(t)\bigr), \phi _{4}\bigl(t,w(t)\bigr),\phi _{5}\bigl(t,w(t)\bigr) \bigr) , $$

hence, the systems of the above integral equations can be written in the following form:

13 $$ w(t)=w_{0}+\frac{2(1-\alpha )}{(2-\alpha )M(\alpha )}\Phi \bigl(t,w(t)-w_{0}\bigr)+\frac{2\alpha }{(2-\alpha )M(\alpha )} \int _{0}^{t}\Phi \bigl(\tau ,w( \tau )\bigr)\,d\tau . $$

We assume the following hypotheses:

$(H_{1})$
There exists a constant $\lambda \in (0,1)$ such that
  $$ \bigl\vert \Phi (t,w)-\Phi (t,\overline{w}) \bigr\vert \leq \lambda \vert w-\overline{w} \vert , $$
  for all $w,\overline{w}\in \mathbb{R}$ and $t\in {}[ 0,T]$.
$(H_{2})$
There exist two positive functions $a,b\in C[0,T]$ such that
  $$ \bigl\vert \Phi (t,w) \bigr\vert \leq a(t)+b(t) \vert w \vert ,\quad \text{for all }t\in {}[ 0,T], w\in \mathbb{R}. $$
  Denote
  $$ M_{0}=\sup_{t\in {}[ 0,T]}a(t),\qquad M_{1}= \sup_{t\in {}[ 0,T]}b(t). $$

Taking equation (13) into account, we define two operators $\mathcal{L}$ and $\mathcal{K}$ as follows:

14 $$\begin{aligned}& \mathcal{L}w ( t ) =w_{0}+ \frac{2(1-\alpha )}{(2-\alpha )M(\alpha )}\Phi \bigl(t,w(t)-w_{0}\bigr), \end{aligned}$$

15 $$\begin{aligned}& \mathcal{K}w ( t ) = \frac{2\alpha }{(2-\alpha )M(\alpha )}\int _{0}^{t}\Phi \bigl(\tau ,w(\tau )\bigr)\,d\tau . \end{aligned}$$

Now, we can state the main theorems of this research study.

### Theorem 3.1

*The problem* (1) *has at least one solution if the hypotheses* $(H_{1})$ *and* $(H_{2})$ *hold and the following condition is satisfied*:

16 $$ \frac{2\lambda (1-\alpha )}{(2-\alpha )M(\alpha )}< 1. $$

### Proof

We use Krasnoselskii’s fixed point theorem (Theorem 2.3).

(i) Let
  $$ X_{\gamma }=\bigl\{ u\in X: \Vert u \Vert \leq \gamma ,\gamma >0\bigr\} , $$
  with
  17 $$ \gamma \geq \vert w_{0} \vert + \frac{2(1-\alpha )}{(2-\alpha )M(\alpha )} \bigl( 2M_{0}+2M_{1} \gamma +M_{1} \vert w_{0} \vert \bigr) , $$
  be a closed and convex set of *X*. Then, for $w,\overline{w}\in $ $X_{\gamma }$, we have
  $$ \begin{aligned} &\bigl\vert \mathcal{L}w ( t ) -\mathcal{L} \overline{w} ( t ) \bigr\vert \\ &\quad = \biggl\vert w_{0}+ \frac{2(1-\alpha )}{(2-\alpha )M(\alpha )}\Phi \bigl(t,w(t)-w_{0}\bigr) - \biggl[ w_{0}+\frac{2(1-\alpha )}{(2-\alpha )M(\alpha )}\Phi \bigl(t,\overline{w}(t)-w_{0}\bigr) \biggr] \biggr\vert \\ &\quad \leq \frac{2(1-\alpha )}{(2-\alpha )M(\alpha )} \bigl\vert \Phi \bigl(t,w(t)-w_{0} \bigr)- \Phi \bigl(t,\overline{w}(t)-w_{0}\bigr) \bigr\vert \\ &\quad \leq \frac{2(1-\alpha )\lambda }{(2-\alpha )M(\alpha )} \bigl\vert w(t)- \overline{w}(t) \bigr\vert , \end{aligned} $$
  hence
  18 $$ \Vert \mathcal{L}w-\mathcal{L}\overline{w} \Vert \leq \frac{2\lambda (1-\alpha )}{(2-\alpha )M(\alpha )} \Vert w- \overline{w} \Vert . $$
  Thanks to inequality (16), the operator $\mathcal{L}$ is a contraction, and then property 2 of Theorem 2.3 is satisfied.
(ii) Now, we have to show that $\mathcal{K}$ is equicontinuous and uniformly bounded. Obviously, $\mathcal{K}$ is continuous, as is Φ, and for all of $w\in X_{\gamma }$ one has
  $$ \bigl\vert \mathcal{K}w ( t ) \bigr\vert \leq \frac{2\alpha }{(2-\alpha )M(\alpha )} \int _{0}^{t} \bigl\vert \Phi \bigl(\tau ,w( \tau )\bigr) \bigr\vert \,d\tau \leq \frac{2\alpha }{(2-\alpha )M(\alpha )} [ M_{0}+M_{1} \gamma ] , $$
  so
  19 $$ \Vert \mathcal{K}w \Vert \leq \frac{2\alpha }{(2-\alpha )M(\alpha )} [ M_{0}+M_{1}\gamma ] . $$
  Thus, from (19), we observe that $\mathcal{K}$ is uniformly bounded.
  To prove the equicontinuity of $\mathcal{K}$, assume $t_{1}< t_{2}\in {}[ 0,T]$, $w\in X_{\gamma }$, then
  20 $$ \begin{aligned} \bigl\vert \mathcal{K}w(t_{1})- \mathcal{K}w(t_{2}) \bigr\vert &= \frac{2\alpha }{(2-\alpha )M(\alpha )} \biggl\vert \int _{0}^{t_{2}} \Phi \bigl(\tau ,w(\tau )\bigr)\,d\tau - \int _{0}^{t_{1}}\Phi \bigl(\tau ,w(\tau )\bigr)\,d\tau \biggr\vert \\ &\leq \frac{2\alpha }{(2-\alpha )M(\alpha )} \biggl\vert \int _{t_{1}}^{t_{2}} \Phi \bigl(\tau ,w(\tau )\bigr)\,d\tau \biggr\vert \\ & \leq \frac{2\alpha }{(2-\alpha )M(\alpha )} ( M_{0}+M_{1}\gamma ) ( t_{2}-t_{1} ) . \end{aligned} $$
  The right-hand side in (20) goes to zero as $t_{1}\rightarrow t_{2}$, therefore, $\mathcal{K}$ is equicontinuous. Hence, by Arzela–Ascoli theorem, $\mathcal{K}$ is relatively compact.
(iii) Let $w,z\in X_{\gamma }$, then
  $$ \begin{aligned} \bigl\vert \mathcal{L}w ( t ) \bigr\vert &= \vert w_{0} \vert + \frac{2(1-\alpha )}{(2-\alpha )M(\alpha )} \bigl\vert \Phi \bigl(t,w(t)-w_{0}\bigr) \bigr\vert \\ &\leq \vert w_{0} \vert + \frac{2(1-\alpha )}{(2-\alpha )M(\alpha )} \bigl( M_{0}+M_{1} \bigl\vert w(t)-w_{0} \bigr\vert \bigr) \\ &\leq \vert w_{0} \vert + \frac{2(1-\alpha )}{(2-\alpha )M(\alpha )} \bigl( M_{0}+M_{1} \bigl( \gamma + \vert w_{0} \vert \bigr) \bigr) . \end{aligned} $$
  Taking (17) and (19) into account, the latter estimate yields
  $$ \begin{aligned} \bigl\vert \mathcal{L}w ( t ) -\mathcal{K}z ( t ) \bigr\vert &\leq \bigl\vert \mathcal{L}w ( t ) \bigr\vert + \bigl\vert \mathcal{K}z ( t ) \bigr\vert \\ &\leq \vert w_{0} \vert + \frac{2(1-\alpha )}{(2-\alpha )M(\alpha )} \bigl( 2M_{0}+2M_{1}\gamma +M_{1} \vert w_{0} \vert \bigr) \leq \gamma. \end{aligned} $$
  Since all statements of Theorem 2.3 are satisfied, the problem (1) has at least one solution.

 □

Now we give the uniqueness result.

### Theorem 3.2

*If the hypotheses* $(H_{1})$ *and* $(H_{2})$ *hold and the following inequality is satisfied*:

21 $$ \frac{2\lambda ( 1-\alpha +\alpha T ) }{(2-\alpha )M(\alpha )}< 1, $$

*then the problem* (1) *has a unique solution*.

### Proof

Define the operator $\mathcal{F}$ on *X* by

22 $$ \begin{aligned} \mathcal{F}w(t)&=w_{0}+\frac{2(1-\alpha )}{(2-\alpha )M(\alpha )}\bigl( \Phi \bigl(t,w(t)\bigr)-\Phi (0,w_{0})\bigr) \\ &\quad {}+ \frac{2\alpha }{(2-\alpha )M(\alpha )}\int _{0}^{t}\Phi \bigl(\tau ,w(\tau )\bigr)\,d\tau . \end{aligned} $$

Let $w,\overline{w}\in X$, then

$$ \begin{aligned} \bigl\vert \mathcal{F}w(t)-\mathcal{F}\overline{w}(t) \bigr\vert &\leq \frac{2(1-\alpha )}{(2-\alpha )M(\alpha )} \bigl\vert \Phi \bigl(t,w(t) \bigr)-\Phi \bigl(t, \overline{w}(t)\bigr) \bigr\vert \\ &\quad{}+\frac{2\alpha }{(2-\alpha )M(\alpha )} \int _{0}^{t} \bigl\vert \Phi \bigl( \tau ,w( \tau )\bigr)-\Phi \bigl(\tau ,\overline{w}(\tau )\bigr) \bigr\vert \,d\tau \\ &\leq \frac{2\lambda ( 1-\alpha +\alpha T ) }{(2-\alpha )M(\alpha )} \Vert w-\overline{w} \Vert . \end{aligned} $$

Hence

23 $$ \Vert \mathcal{F}w-\mathcal{F}\overline{w} \Vert \leq \frac{2\lambda ( 1-\alpha +\alpha T ) }{(2-\alpha )M(\alpha )} \Vert w- \overline{w} \Vert . $$

From inequality (23), we observe that $\mathcal{F}$ is a contraction, thus from Banach’s contraction principle we conclude that $\mathcal{F}$ has a unique fixed point, which is the unique solution of equation (13), and thus problem (1) has a unique solution. □

Again, in the following theorems, we can investigate the solution to problem (1) by using another type of fixed point theorem known as Schauder’s fixed point theorem.

### Theorem 3.3

*Assume that*

24 $$ T< \biggl( \frac{2(1-\alpha )M(\alpha )}{5\max ( (\omega +\nu ), ( \nu +(1-\varepsilon )\mu ) , ( \eta +\omega ) , ( \nu +\eta ) ,\overline{\omega } ) }+ \frac{\alpha -1}{\alpha } \biggr) . $$

*Then*, *the problem* (1) *has at least one solution*.

### Proof

Set

$$ \mathcal{F}_{i}u(t)=u_{0}+\frac{2(1-\alpha )}{(2-\alpha )M(\alpha )}\bigl( \phi _{i}\bigl(t,u(t)\bigr)-\phi _{i}\bigl(0,u(0)\bigr) \bigr)+ \frac{2\alpha }{(2-\alpha )M(\alpha )}\int _{0}^{t}\phi _{i}\bigl(\tau ,u(\tau )\bigr)\,d\tau , $$

then

$$ \mathcal{F}u(t)= \bigl( \mathcal{F}_{1}u(t),\mathcal{F}_{2}u(t), \mathcal{F}_{3}u(t),\mathcal{F}_{4}u(t), \mathcal{F}_{5}u(t) \bigr) . $$

(i) Operator $\mathcal{F}$ is continuous. In fact, for the sequence $u_{n}= ( \mathcal{S}_{n},\mathcal{V}_{n},\mathcal{I}_{n},\mathcal{R}_{n},\mathcal{D}_{n} ) $ such that $\lim_{n\rightarrow \infty }u_{n}=u$ we have, for all $t\in {}[ 0,T]$,

25 $$\begin{aligned} &\bigl\vert \mathcal{F}_{i}u_{n}(t)- \mathcal{F}_{i}u(t) \bigr\vert \\ &\quad = \biggl\vert u_{0}+ \frac{2(1-\alpha )}{(2-\alpha )M(\alpha )}\bigl(\phi _{i} \bigl(t,u_{n}(t)\bigr)-\phi _{i}\bigl(0,u(0)\bigr)\bigr) \\ &\quad \quad{}+\frac{2\alpha }{(2-\alpha )M(\alpha )} \int _{0}^{t}\phi _{i}\bigl( \tau ,u_{n}(\tau )\bigr)\,d\tau \biggr\vert \\ &\quad \quad{}- \biggl[ u_{0}+\frac{2(1-\alpha )}{(2-\alpha )M(\alpha )}\bigl( \phi _{i}\bigl(t,u(t)\bigr)-\phi _{i}\bigl(0,u(0)\bigr) \bigr) \\ &\quad \quad {}+ \frac{2\alpha }{(2-\alpha )M(\alpha )}\int _{0}^{t}\phi _{i}\bigl(\tau ,u(\tau )\bigr)\,d\tau \biggr] \\ &\quad = \biggl\vert \frac{2(1-\alpha )}{(2-\alpha )M(\alpha )}\phi _{i} \bigl(t,u_{n}(t)-u(t)\bigr)+ \frac{2\alpha }{(2-\alpha )M(\alpha )} \int _{0}^{t}\phi _{i}\bigl(\tau ,u_{n}( \tau )-u(\tau )\bigr)\,d\tau \biggr\vert \\ &\quad \leq \frac{2(1-\alpha )}{(2-\alpha )M(\alpha )} \bigl\vert \phi _{i} \bigl(t,u_{n}(t)-u(t)\bigr) \bigr\vert +\frac{2\alpha }{(2-\alpha )M(\alpha )} \int _{0}^{t} \bigl\vert \phi _{i}\bigl(\tau ,u_{n}( \tau )-u(\tau )\bigr)\,d\tau \bigr\vert , \end{aligned}$$

where $\phi _{i}$ satisfies (12) for each $1\leq i\leq 5$. This implies that $\phi _{i}(t,u_{n}(t))\rightarrow \phi _{i}(t,u(t))$ in $[0,T]\times \mathbb{R}^{5}$. In fact, we have

$$ \begin{aligned} &\bigl\vert \phi _{1}\bigl(t,u_{n}(t) \bigr)-\phi _{1}\bigl(t,u(t)\bigr) \bigr\vert \\ &\quad = \bigl\vert \bigl( - ( \omega +\nu ) \mathcal{S}_{n}(t)-\mu \mathcal{I}_{n}(t) \mathcal{S}_{n}(t) \bigr) - \bigl( - ( \omega +\nu ) \mathcal{S}(t)- \mu \mathcal{I}(t)\mathcal{S}(t) \bigr) \bigr\vert \\ &\quad \leq ( \omega +\nu ) \bigl\vert \mathcal{S}_{n}(t)- \mathcal{S}(t) \bigr\vert + \mu \bigl\vert \mathcal{I}_{n}(t) \mathcal{S}_{n}(t)-\mathcal{I}(t)\mathcal{S}(t) \bigr\vert \\ &\quad \leq \bigl\{ \bigl( \omega +\nu +\mu \mathcal{I}_{n}(t) \bigr) \bigl\vert \mathcal{S}_{n}(t)-\mathcal{S}(t) \bigr\vert +\mu \mathcal{S}(t) \bigl\vert \mathcal{I}_{n}(t)-\mathcal{I}(t) \bigr\vert \bigr\} \\ &\quad \leq \bigl\{ \omega +\nu +\mu \bigl( \Vert \mathcal{I}_{n} \Vert + \Vert \mathcal{S} \Vert \bigr) \bigr\} \Vert u_{n}-u \Vert . \end{aligned} $$

Then,

26 $$ \begin{aligned} &\bigl\vert \mathcal{F}_{1}u_{n}(t)- \mathcal{F}_{1}u(t) \bigr\vert \\ &\quad \leq \biggl( \frac{2(1-\alpha )}{(2-\alpha )M(\alpha )}+ \frac{2\alpha T}{(2-\alpha )M(\alpha )} \biggr) \bigl\{ \omega +\nu + \mu \bigl( \Vert \mathcal{I}_{n} \Vert + \Vert \mathcal{S} \Vert \bigr) \bigr\} \Vert u_{n}-u \Vert \\ &\quad \rightarrow 0,\quad \text{ as }n\rightarrow \infty . \end{aligned} $$

Similarly, we show that

$$ \bigl\vert \mathcal{F}_{i}u_{n}(t)- \mathcal{F}_{i}u(t) \bigr\vert \rightarrow 0,\quad \text{as }n\rightarrow \infty ,\ i=2,3,4,5, $$

and hence,

$$ \lim_{n\rightarrow \infty } \Vert \mathcal{F}u_{n}- \mathcal{F}u \Vert =0, $$

implying the continuity of $\mathcal{F}$.

(ii) Define the set *B* as a subset of *X* given by

$$ B=\bigl\{ u\in X: \Vert u \Vert \leq \xi \bigr\} , $$

where *ξ* satisfies

27 $$ \xi \geq \frac{10(1-\alpha +\alpha T)}{(2-\alpha )M(\alpha )} \bigl( \max \bigl( (\omega +\nu ), \bigl( \nu +(1-\varepsilon )\mu \bigr) , ( \eta +\omega ) , ( \nu +\eta ) , \overline{\omega } \bigr) \xi +\mu \xi ^{2} \bigr) . $$

Then, for all $t\in {}[ 0,T]$ we have

$$\begin{aligned} & \bigl\vert \phi _{1}\bigl(t,u(t)\bigr) \bigr\vert = \bigl\vert - ( \omega +\nu ) \mathcal{S}-\mu \mathcal{I}\mathcal{S} \bigr\vert \leq ( \omega +\nu ) \Vert \mathcal{S} \Vert +\mu \Vert \mathcal{I} \Vert \Vert \mathcal{S} \Vert \leq ( \omega +\nu ) \xi +\mu \xi ^{2}, \\ & \bigl\vert \phi _{2}\bigl(t,u(t)\bigr) \bigr\vert = \bigl\vert \nu \mathcal{S}-(1-\varepsilon )\mu \mathcal{V} \bigr\vert \leq (\nu ) \Vert \mathcal{S} \Vert +(1- \varepsilon )\mu \Vert \mathcal{V} \Vert \leq \bigl( \nu +(1-\varepsilon )\mu \bigr) \xi , \\ & \bigl\vert \phi _{3}\bigl(t,u(t)\bigr) \bigr\vert = \bigl\vert \mu \mathcal{S}\mathcal{I}-(\eta +\omega ) \mathcal{I} \bigr\vert \leq \mu \Vert \mathcal{S} \Vert \Vert \mathcal{I} \Vert +(\eta +\omega ) \Vert \mathcal{I} \Vert \leq \mu \xi ^{2}+ ( \eta +\omega ) \xi , \\ & \bigl\vert \phi _{4}\bigl(t,u(t)\bigr) \bigr\vert = \vert \nu \mathcal{S}+\eta \mathcal{I} \vert \leq \nu \Vert S \Vert +\eta \Vert \mathcal{I} \Vert \leq ( \nu +\eta ) \xi , \\ & \bigl\vert \phi _{5}\bigl(t,u(t)\bigr) \bigr\vert = \vert \overline{\omega }\mathcal{I} \vert \leq \overline{\omega } \Vert \mathcal{I} \Vert \leq \overline{\omega }\xi . \end{aligned}$$

Thus

28 $$ \bigl\vert \phi _{i}\bigl(t,u(t)\bigr) \bigr\vert \leq \max \bigl( (\omega +\nu ), \bigl( \nu +(1- \varepsilon )\mu \bigr) , ( \eta +\omega ) , ( \nu +\eta ) ,\overline{\omega } \bigr) \xi +\mu \xi ^{2}. $$

As a result of (28), we can conclude that

29 $$ \begin{aligned} &\xi \geq \frac{10(1-\alpha +\alpha T)}{(2-\alpha )M(\alpha )} \bigl( \max \bigl( ( \omega +\nu ), \bigl( \nu +(1-\varepsilon )\mu \bigr) , ( \eta +\omega ) , ( \nu + \eta ) , \overline{\omega } \bigr) \xi +\mu \xi ^{2} \bigr) , \\ & \begin{aligned} &\bigl|\mathcal{F}_{i}u(t))\bigr| \\ &\quad \leq \frac{2(1-\alpha +\alpha T)}{(2-\alpha )M(\alpha )} \bigl( \max \bigl( (\omega +\nu ), \bigl( \nu +(1-\varepsilon )\mu \bigr) , ( \eta +\omega ) , ( \nu +\eta ) , \overline{\omega } \bigr) \xi +\mu \xi ^{2} \bigr) \\ &\quad \leq \frac{\xi }{5}. \end{aligned} \end{aligned} $$

Thus

$$ \Vert \mathcal{F}u \Vert =\sum_{i=1}^{5} \Vert \mathcal{F}_{i}u \Vert \leq \xi . $$

Consequently, $\mathcal{F}(B)\subset B$.

(iii) We show that $\mathcal{F}(B)$ is relatively compact. Let $t_{1},t_{2}\in {}[ 0,T]$, $t_{1}< t_{2}$ and $u\in B$. Then, for all $i=1,2,3,4,5$, we get

$$ \begin{aligned} & \bigl\vert \mathcal{F}_{i}u(t_{2})- \mathcal{F}_{i}u(t_{1}) \bigr\vert \\ &\quad = \biggl\vert \biggl( u_{0}+\frac{2(1-\alpha )}{(2-\alpha )M(\alpha )} \bigl[ \phi _{i}\bigl(t_{2},u(t_{2})\bigr)- \phi _{i}\bigl(0,u(0)\bigr) \bigr] \\ &\quad \quad {}+\frac{2\alpha }{(2-\alpha )M(\alpha )}\int _{0}^{t_{2}}\phi _{i}\bigl(\tau ,u(\tau )\bigr)\,d\tau \biggr) \\ &\quad \quad{}- \biggl( u_{0}+\frac{2(1-\alpha )}{(2-\alpha )M(\alpha )} \bigl[ \phi _{i}\bigl(t_{1},u(t_{1})\bigr)-\phi _{i}\bigl(0,u(0)\bigr) \bigr] \\ &\quad \quad {} + \frac{2\alpha }{(2-\alpha )M(\alpha )} \int _{0}^{t_{1}}\phi _{i}\bigl( \tau ,u(\tau )\bigr)\,d\tau \biggr) \biggr\vert \\ &\quad \leq \biggl\vert \frac{2(1-\alpha )}{(2-\alpha )M(\alpha )} \bigl[ \phi _{i} \bigl(t_{2},u(t_{2})\bigr)-\phi _{i} \bigl(t_{1},u(t_{1})\bigr) \bigr] \biggr\vert \\ &\quad \quad{}+\frac{2\alpha }{(2-\alpha )M(\alpha )} \biggl\vert \int _{0}^{t_{2}} \phi _{i}\bigl(\tau ,u(\tau )\bigr)\,d\tau - \int _{0}^{t_{1}}\phi _{i}\bigl(\tau ,u( \tau )\bigr)\,d\tau \biggr\vert \\ &\quad \leq \frac{2(1-\alpha )}{(2-\alpha )M(\alpha )}\lambda\bigl\vert u(t_{2})-u(t_{1}) \bigr\vert +\frac{2\alpha }{(2-\alpha )M(\alpha )}\int _{t_{1}}^{t_{2}} \bigl\vert \phi _{i}\bigl(\tau ,u(\tau )\bigr)\bigr\vert \,d\tau \\ &\quad \leq \frac{2(1-\alpha )\lambda }{(2-\alpha )M(\alpha )}\bigl\vert u(t_{2})-u(t_{1}) \bigr\vert \\ &\quad \quad{}+\frac{2\alpha }{(2-\alpha )M(\alpha )} \bigl( \max \bigl( ( \omega +\nu ), \bigl( \nu +(1-\varepsilon )\mu \bigr) , ( \eta +\omega ) , ( \nu +\eta ) ,\overline{\omega } \bigr) \xi +\mu \xi ^{2} \bigr) ( t_{2}-t_{1} ) . \end{aligned} $$

The right-hand side of the above inequality tends to zero if $t_{1}\rightarrow t_{2}$, $\forall i=1,2,3,4,5$. Therefore, from Ascoli–Arzela theorem and steps (i) to (iii), we observe that $\mathcal{F}$ is relatively compact. Since all the conditions of Schauder’s fixed point theorem are satisfied, operator $\mathcal{F}$ has at least one fixed point, which is a solution for the problem (1). □

We give a uniqueness theorem under another hypothesis.

### Theorem 3.4

*Assume that the following inequality holds*:

30 $$ \biggl[ \frac{10(1-\alpha +\alpha T)\delta }{(2-\alpha )M(\alpha )} \biggr] < 1, $$

*where*

$$ \delta =\max \bigl\{ \omega +\nu +2\mu ,\nu + ( 1-\varepsilon ) \mu ,2\mu +\eta + \omega ,\nu +\eta ,\overline{\omega }\bigr\} , $$

*then the problem* (1) *has a unique solution*.

### Proof

Let $u= ( \mathcal{S},\mathcal{V},\mathcal{I},\mathcal{R},\mathcal{D} ) $ and $\widetilde{u}= ( \widetilde{\mathcal{S}},\widetilde{\mathcal{V}},\widetilde{\mathcal{I}}, \widetilde{\mathcal{R}},\widetilde{\mathcal{D}} ) $ be two solutions for the problem (1). Then for each $t\in {}[ 0,T]$, we have

$$ \begin{aligned} \bigl\vert \mathcal{F}_{i}u(t)- \mathcal{F}_{i}\widetilde{u}(t) \bigr\vert & \leq \frac{2(1-\alpha )}{(2-\alpha )M(\alpha )} \bigl\vert \phi _{i}\bigl(t,u(t)\bigr)-\phi _{i}\bigl(t,\widetilde{u}(t)\bigr) \bigr\vert \\ &\quad{}+\frac{2\alpha }{(2-\alpha )M(\alpha )} \int _{0}^{t} \bigl\vert \phi _{i}\bigl( \tau ,u(\tau )\bigr)-\phi _{i}\bigl(\tau , \widetilde{u}(\tau )\bigr) \bigr\vert \,d\tau . \end{aligned} $$

Particularly, we obtain

$$ \begin{aligned} \bigl\vert \phi _{1}\bigl(t,u(t)\bigr)- \phi _{1}\bigl(t,\widetilde{u}(t)\bigr) \bigr\vert &\leq ( \omega + \nu ) \bigl\vert S(t)-\widetilde{\mathcal{S}}(t) \bigr\vert +\mu \bigl\vert \mathcal{I}(t)\mathcal{S}(t)-\widetilde{\mathcal{I}}(t)\widetilde{ \mathcal{S}}(t) \bigr\vert \\ & \leq \max_{{}[ 0,T]}\bigl\{ ( \omega +\nu ) \bigl\vert S(t)- \widetilde{\mathcal{S}}(t) \bigr\vert +\mu \mathcal{I}(t) \bigl\vert \mathcal{S}(t)- \widetilde{\mathcal{S}}(t) \bigr\vert \\ &\quad{}+\mu \widetilde{\mathcal{S}}(t) \bigl\vert \mathcal{I}(t)- \widetilde{ \mathcal{I}}(t) \bigr\vert \bigr\} \\ & \leq \max_{{}[ 0,T]}\bigl\{ \bigl( \omega +\nu +\mu \bigl\vert \mathcal{I}(t) \bigr\vert \bigr) \bigl\vert u(t)-\widetilde{u}(t) \bigr\vert +\mu \bigl\vert \widetilde{\mathcal{S}}(t) \bigr\vert \bigl\vert u(t)- \widetilde{u}(t) \bigr\vert \bigr\} \\ & \leq \{\omega +\nu +2\mu \} \Vert u-\widetilde{u} \Vert . \end{aligned} $$

Similarly, we get

$$\begin{aligned}& \bigl\vert \phi _{2}\bigl(t,u(t)\bigr)-\phi _{2} \bigl(t,\widetilde{u}(t)\bigr) \bigr\vert \leq \bigl\{ \nu + ( 1- \varepsilon ) \mu \bigr\} \Vert u-\widetilde{u} \Vert , \\& \bigl\vert \phi _{3}\bigl(t,u(t)\bigr)-\phi _{3} \bigl(t,\widetilde{u}(t)\bigr) \bigr\vert \leq \bigl\{ 2\mu + ( \eta +\omega ) \bigr\} \Vert u-\widetilde{u} \Vert , \\& \bigl\vert \phi _{4}\bigl(t,u(t)\bigr)-\phi _{4} \bigl(t,\widetilde{u}(t)\bigr) \bigr\vert \leq ( \nu + \eta ) \Vert u- \widetilde{u} \Vert , \\& \bigl\vert \phi _{5}\bigl(t,u(t)\bigr)-\phi _{5} \bigl(t,\widetilde{u}(t)\bigr) \bigr\vert \leq \overline{\omega } \Vert u-\widetilde{u} \Vert . \end{aligned}$$

Then

$$ \bigl\vert \phi _{i}\bigl(t,u(t)\bigr)-\phi _{i} \bigl(t,\widetilde{u}(t)\bigr) \bigr\vert \leq \delta \Vert u-\widetilde{u} \Vert ,\quad \forall i=1,2,3,4,5. $$

Hence

$$ \Vert \mathcal{F}u-\mathcal{F}\widetilde{u} \Vert \leq \biggl[ \frac{10(1-\alpha )+\alpha T)\delta }{(2-\alpha )M(\alpha )} \biggr] \Vert u-\widetilde{u} \Vert ,\quad \forall i=1,2,3,4,5, $$

thus $\mathcal{F}$ is a contraction. We conclude that $\mathcal{F}$ has a unique point as a consequence of the Banach contraction principle, which is the unique solution for the problem (1). □

Let us study the Ulam–Hyers stability of the considered problem.

### Definition 3.1

The problem (1) is Ulam–Hyers stable if for any $\epsilon >0$ such that the inequality

31 $$ \bigl\vert {}^{\mathcal{CF}}D_{0^{+}}^{\alpha }w(t)- \Phi \bigl(t,w(t)\bigr) \bigr\vert \leq \epsilon $$

holds, there exist a constant $c>0$ and a unique solution *z* of problem (1) such

$$ \Vert w-z \Vert \leq c\epsilon . $$

### Remark 3.1

If inequality (31) holds then there exists a function *ϰ* depending on $w\in X$ such that $\varkappa ( 0 ) =0$, $\vert \varkappa ( t ) \vert \leq \epsilon $ for all $t\in [ 0,T ] $, and

32 $$ {}^{\mathcal{CF}}D_{0^{+}}^{\alpha }w(t)=\Phi \bigl(t,w(t)\bigr)+ \varkappa ( t ) ,\quad t\in [ 0,T ]. $$

### Lemma 3.1

*The solution of the problem*

33 $$\begin{aligned}& {}^{\mathcal{CF}}D_{0^{+}}^{\alpha }w(t) =\Phi \bigl(t,w(t) \bigr)+\varkappa ( t ) ,\quad t\in [ 0,T ], \end{aligned}$$

34 $$\begin{aligned}& w(0) =w_{0} \end{aligned}$$

*is*

$$ \begin{aligned} w(t) &=w_{0}+\frac{2(1-\alpha )}{(2-\alpha )M(\alpha )}\bigl( \phi \bigl(t,w(t)\bigr)-\phi (0,w_{0})\bigr)+\frac{2\alpha }{(2-\alpha )M(\alpha )} \int _{0}^{t}\phi \bigl(\tau ,w(\tau )\bigr)\,d\tau \\ &\quad{}+\frac{2(1-\alpha )}{(2-\alpha )M(\alpha )}\varkappa ( t ) +\frac{2\alpha }{(2-\alpha )M(\alpha )} \int _{0}^{t}\varkappa (\tau )\,d\tau . \end{aligned} $$

*Moreover*, *the solution satisfies the following inequality*:

35 $$ \begin{aligned} &\biggl\vert w(t)-[w_{0}+\frac{2(1-\alpha )}{(2-\alpha )M(\alpha )}\bigl( \phi \bigl(t,w(t) \bigr)-\phi (0,w_{0})\bigr) \\ &\quad \quad {}+\frac{2\alpha }{(2-\alpha )M(\alpha )} \int _{0}^{t}\phi \bigl(\tau ,w(\tau )\bigr)\,d\tau \biggr\vert \\ &\quad \leq \frac{2(1-\alpha +\alpha T)}{(2-\alpha )M(\alpha )}\epsilon . \end{aligned} $$

### Theorem 3.5

*The problem* (1) *is Ulam–Hyers stable if the hypotheses* $(H_{1})$ *and* $(H_{2}) $ *are satisfied and the following inequality holds*:

36 $$ \frac{(2-\alpha +\alpha T)M(\alpha )}{(2-\alpha )M(\alpha )-2\delta (1-\alpha +\alpha T)}< 1. $$

### Proof

Let *u* be a solution of inequality (31) and *ũ* be a solution of problem (1), then we have

$$ \begin{aligned} \Vert u-\widetilde{u} \Vert &=\max _{t\in {}[ 0,T]} \biggl\vert u(t)- \biggl[ \widetilde{u}_{0}+ \frac{2(1-\alpha )}{(2-\alpha )M(\alpha )}\bigl(\phi (t,\widetilde{u})-\phi (0, \widetilde{u}_{0})\bigr) \\ &\quad {}+ \frac{2\alpha }{(2-\alpha )M(\alpha )} \int _{0}^{t}\bigl(\phi \bigl(\tau , \widetilde{u}(\tau )\bigr)\bigr)\,d\tau \biggr] \biggr\vert \\ &\leq \max_{t\in {}[ 0,T]}\biggl\vert u- \biggl[ u_{0}+ \frac{2(1-\alpha )}{(2-\alpha )M(\alpha )}\bigl(\phi (t,u)-\phi (0,u_{0})\bigr) \\ &\quad {}+ \frac{2\alpha }{(2-\alpha )M(\alpha )} \int _{0}^{t}\bigl(\phi \bigl(\tau ,u( \tau )\bigr)\bigr)\,d\tau \biggr] \biggr\vert \\ &\quad{}+\frac{2(1-\alpha )}{(2-\alpha )M(\alpha )}\max_{t\in {}[ 0,T]} \bigl\{ \bigl\vert \phi (t,u)-\phi (t,\widetilde{u}) \bigr\vert \bigr\} \\ &\quad{}+\frac{2\alpha }{(2-\alpha )M(\alpha )}\max_{t\in {}[ 0,T]} \biggl\{ \int _{0}^{t} \bigl\vert \phi \bigl(\tau ,u( \tau )\bigr)-\phi \bigl(\tau ,\widetilde{u}(\tau )\bigr) \bigr\vert \,d\tau \biggr\} . \end{aligned} $$

Since

$$ \bigl\vert \phi \bigl(t,u(t)\bigr)-\phi \bigl(t,\widetilde{u}(t)\bigr) \bigr\vert \leq \delta \Vert u- \widetilde{u} \Vert , $$

we get

37 $$ \Vert u-\widetilde{u} \Vert \leq \frac{2(1-\alpha +\alpha T)}{(2-\alpha )M(\alpha )} \epsilon + \frac{2\delta (1-\alpha +\alpha T)}{(2-\alpha )M(\alpha )} \Vert u- \tilde{u} \Vert . $$

From (37) we get

38 $$ \Vert u-\tilde{u} \Vert \leq \frac{(2-\alpha +\alpha T)M(\alpha )}{(2-\alpha )M(\alpha )-2\delta (1-\alpha +\alpha T)} \epsilon . $$

From inequality (38), we deduce that the solution of problem (1) is Ulam–Hyers stable. □

## Conclusions

We investigated the solution of the developed mathematical model (1) of COVID-19 involving nonlinear Caputo–Fabrizio fractional derivative systems in this study. First, we have constructed an existence, uniqueness, and stability criterion with the aid of the Lipschitz condition to analyze the solution of a generated model (1). We have transformed the proposed mathematical model (1) to a fixed point problem, and the existence of a solution was investigated based on the arguments of the Krasnoselskii’s and Schauder’s fixed point theorems. Also, we have examined the uniqueness of the solution to the given problem by employing the Banach contraction principle, then the stability of the solution has been proved by using the Ulam–Hyers stability theorem. We have determined the significance and effectiveness of fixed point theorems in the qualitative analysis of solutions to systems of nonlinear fractional differential equations. This study can be extended to more general epidemic models involving other types of fractional derivative and using numerical methods.

## Data Availability

Not applicable.
